# Transitional fracture of the distal radius: a rare injury in adolescent athletes. Case series and literature review

**DOI:** 10.1186/s40001-020-00419-0

**Published:** 2020-06-09

**Authors:** Thomas Rauer, Hans-Christoph Pape, Jamison G. Gamble, Nicolo’ Vitale, Sascha Halvachizadeh, Florin Allemann

**Affiliations:** 1 Division of Trauma Surgery, UniversityHospital Zurich, Rämistrasse 100, 8091 Zurich, Switzerland; 2 St. George’s University School of Medicine, St. George, Grenada; 3 Physical Medicine and Rehabilitation, Department of Biomedicine and Biotechnology, University of Catania, Catania, Italy

**Keywords:** Distal radius fracture, Transitional fracture, Fracture treatment, Adolescent, Physeal plate of the distal radius

## Abstract

**Background:**

Transitional fractures are fractures in adolescents where partial closure of the epiphyseal growth plate has occurred. These fractures are most commonly reported in the distal tibia. With respect to the distal radius, only a few case reports describing transitional fractures exist. Furthermore, relatively little is known about epiphyseal closure of the distal radius. A case series of four transitional fractures of the distal radius is presented by comparing non-operative and operative treatment options. At present, this is the largest case series in the literature dealing with this rare injury.

**Case presentation:**

We present three cases of four transitional fractures of the distal radius including 1–year follow-up. Patient age ranged from 16 to 18 years including a gender ratio of two males to one female. Clinical and radiographic assessments took place 6 and 12 weeks and 1 year after trauma/surgery. Three transitional fractures were treated with open reduction and internal volar plate fixation followed by functional rehabilitation. One transitional fracture was treated non-operatively. All cases showed an excellent functional outcome.

**Conclusions:**

The primary treatment goal in transitional fractures is anatomic reduction of the articular surface. Non-operative treatment of transitional fractures of the distal radius is the most commonly reported treatment option. Additionally, different fixation options have been described, including the use of Kirschner wires (K-wires) and lag screws. The presented cases demonstrate that volar plate fixation followed by functional rehabilitation is a valuable treatment option in significantly displaced transitional fractures of the distal radius. Furthermore, we discuss the pathogenesis as well as the different treatment options by critical reviewing the literature.

## Background

Distal radius fractures are one of the most common human osseous injuries, with incidence rates increasing worldwide [1–6]. There are two peaks of prevalence: the first around the 10th and the second around the 60th year of life [1]. During childhood, they are among the most common pediatric fractures [7] accounting for 19.9 to 35.8% of all pediatric fractures [8, 9]. Transitional fractures are defined as epiphyseal injuries in which partial closure of the epiphyseal growth plate has already occurred [10] and must be clearly differentiated from pediatric fractures as an independent fracture entity. Despite the frequency of distal radius fractures, especially during childhood, transitional fractures of the distal radius are rare and up until today only a few case reports have been published [11–16]. A case series of four transitional fractures of the distal radius with discussion of the diagnosis and treatment options and critical review of the literature is presented here. The case series presented here is the largest in the current literature.

## Case presentation

Our study considered three cases of four transitional fractures of the distal radius including 1-year follow-up. Patient age ranged from 16 to 18 years including a gender ratio of two males to one female. Clinical and radiographic assessments took place 6 and 12 weeks and 1 year after trauma/surgery. Three months after trauma/surgery the Quick DASH (Disabilities of the Arm, Shoulder, Hand) Score was administered. Informed consent was obtained from all patients included in this case series. This study was approved by the institutional review board (business administration system for ethics committees, BASEC, No. 2018-00146).

### Case I

**Fig. 1:**
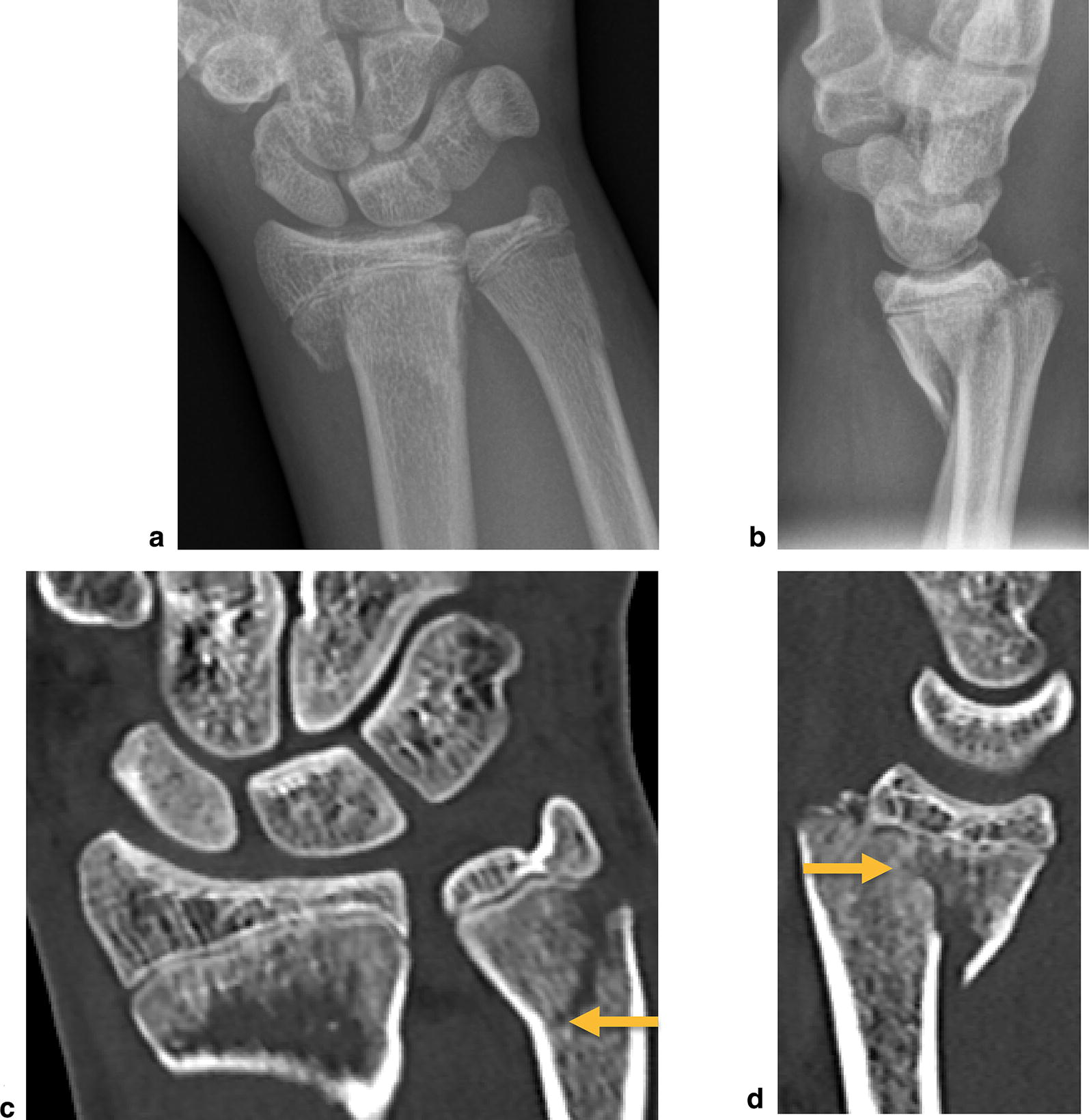
Diagnostic imaging case 1. Radiograph (**a** antero-posterior view; **b** lateral view) and CT scan (**c** coronal view, arrow indicating transitional fracture of the distal ulna; **d** sagittal view, arrow indicating transitional fracture of the distal radius) of the right wrist, showing a transitional both of the distal radius and the distal ulna

**Fig. 2:**
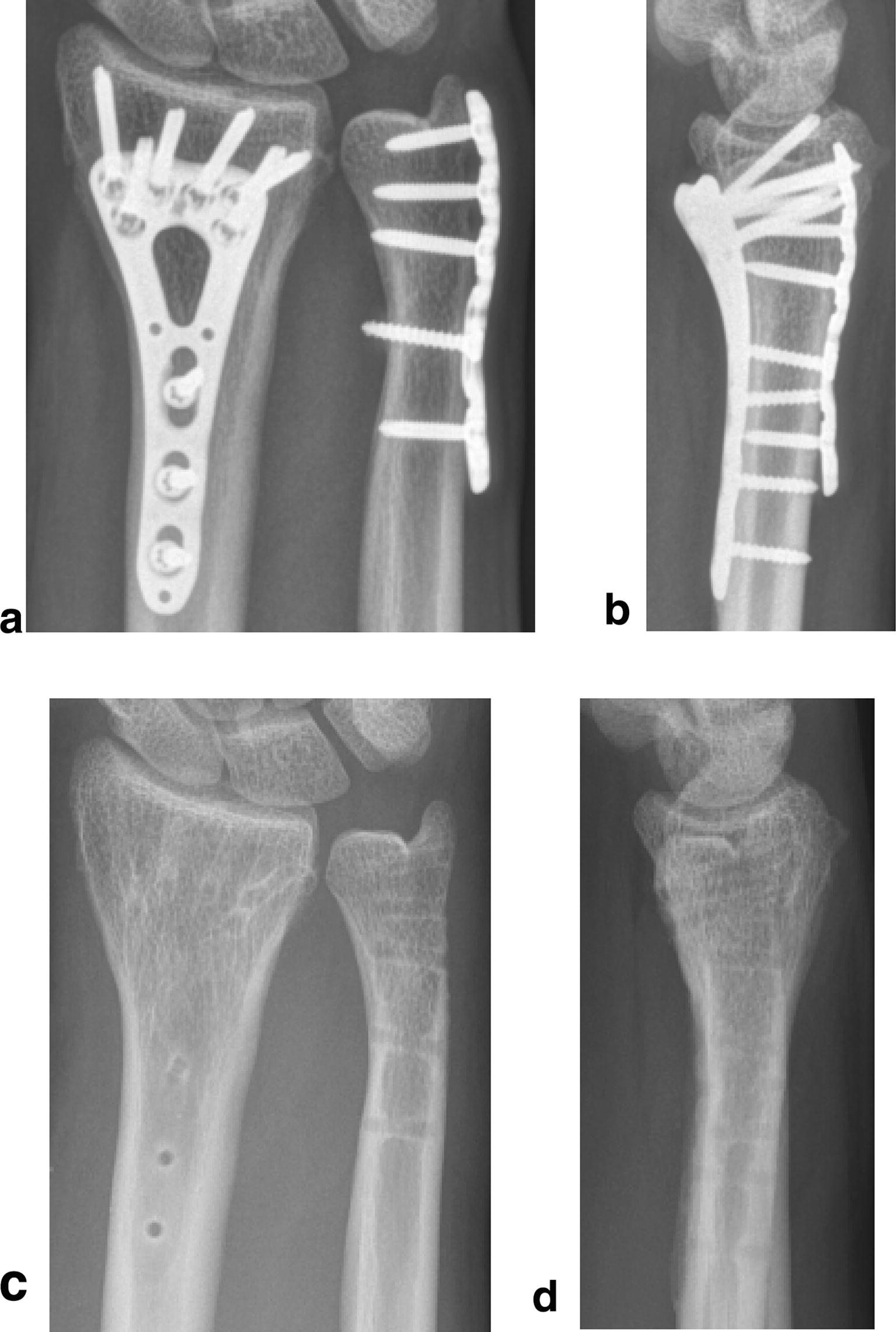
Follow-up imaging case 1. Radiograph (**a** antero-posterior view; **b** lateral view) demonstrating healed fractures 3 months postoperatively and radiograph (**c** antero-posterior view; **d** lateral view) demonstrating healed fractures after removal of the osteosynthesis after 1 year

A healthy 16-year-old man was admitted to the emergency department after falling off his skateboard onto his right hand. Swelling and hematoma with local tenderness was present around the right wrist without neurovascular impairment. Radiograph and the subsequent CT scan demonstrated a displaced transitional fracture both of the distal radius and the distal ulna (Fig. 1). An open reduction and internal plate fixation of both fractures was performed. The transitional fracture of the distal radius was treated through a volar approach (modified Henry approach): a skin incision along the radial border of the flexor carpi radialis tendon was done. The flexor tendons were retracted and the pronator quadratus muscle was exposed. The pronator quadratus muscle was released on its radial border and lifted by subperiosteal dissection to expose the fracture. Preliminary anatomical reduction of the radial fracture was achieved by direct manipulation of the fracture and secured with a volar 1.2-mm K-wire. Under observation from the image intensifiers, a Variable Angle LCP Two-Column Volar Distal Radius Plate 2.4 mm (DePuy Synthes, CH-4528 Zuchwil, Switzerland) was attached to the distal radial shaft ending at the anatomic watershed zone of the distal radius. The transitional fracture of the distal ulna was then treated through an ulnar approach: a longitudinal skin incision was made over the distal ulna and the interval between the tendons of the extensor and flexor carpi ulnaris was developed to expose the fracture. Preliminary anatomical reduction of the ulna fracture was achieved by direct manipulation of the fracture and secured with a 1.2-mm K-wire. Under observation from the image intensifiers a Variable Angle LCP Dorsal Distal Radius Plate 2.4 mm (DePuy Synthes, CH-4528 Zuchwil, Switzerland) was attached to the distal ulna. Three months postoperatively, following functional rehabilitation without cast immobilization, the patient demonstrated bilateral equal range of motion of the wrist. Radiographic examination showed complete healing of the fractures (Fig. 2a, b), at which point in time administration of the Quick DASH (Disabilities of the Arm, Shoulder, Hand) revealed a score of 11.4. Removal of osteosynthesis material was made after 1 year (Fig. 2c, d).

### Case II

**Fig. 3:**
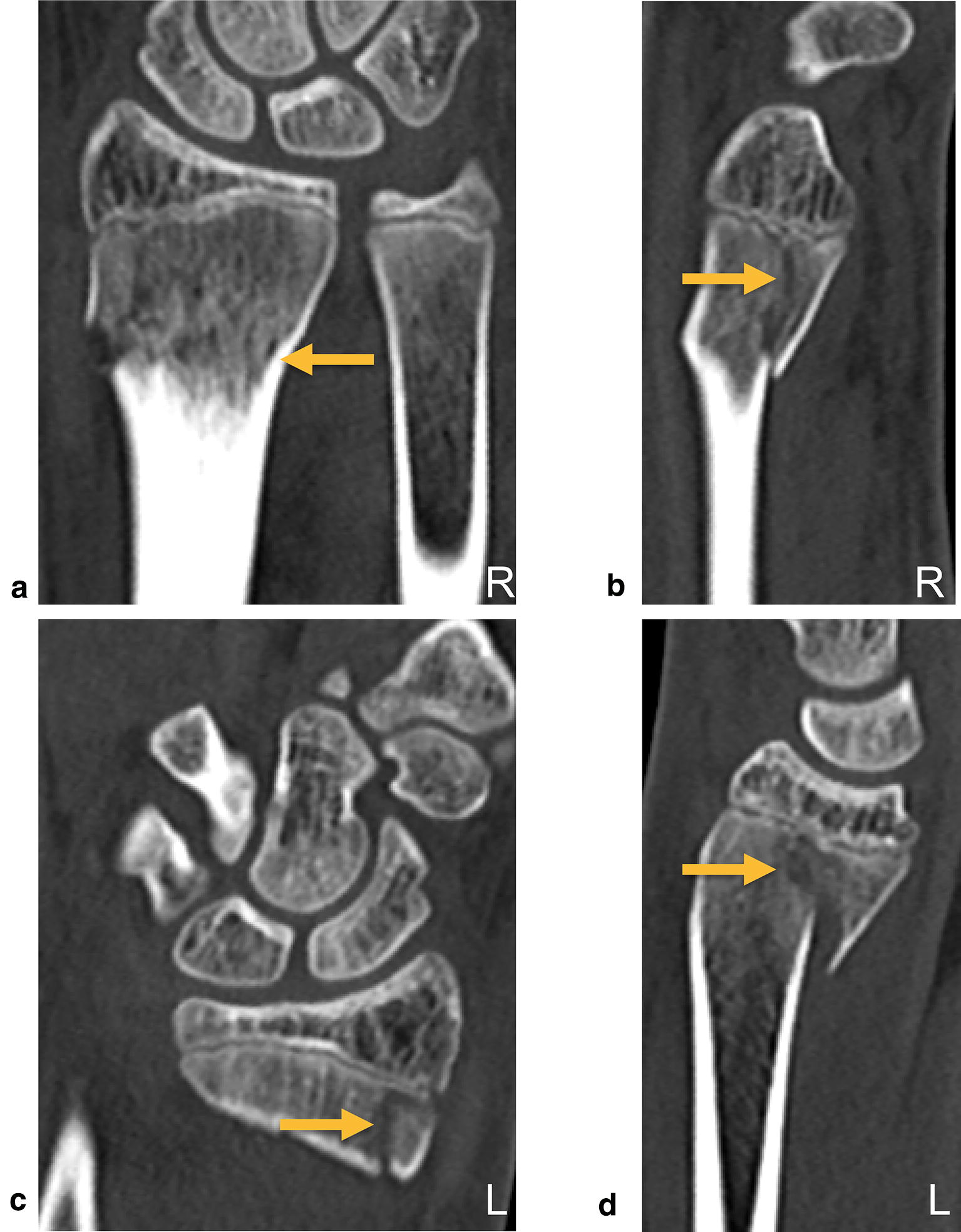
Diagnostic imaging case 2, CT scan (**a** coronal view right side, **b** sagittal view right side, **c** coronal view left side, **d** sagittal view left side) demonstrating a displaced transitional fracture of the distal radius on both sides (arrows)

**Fig. 4:**
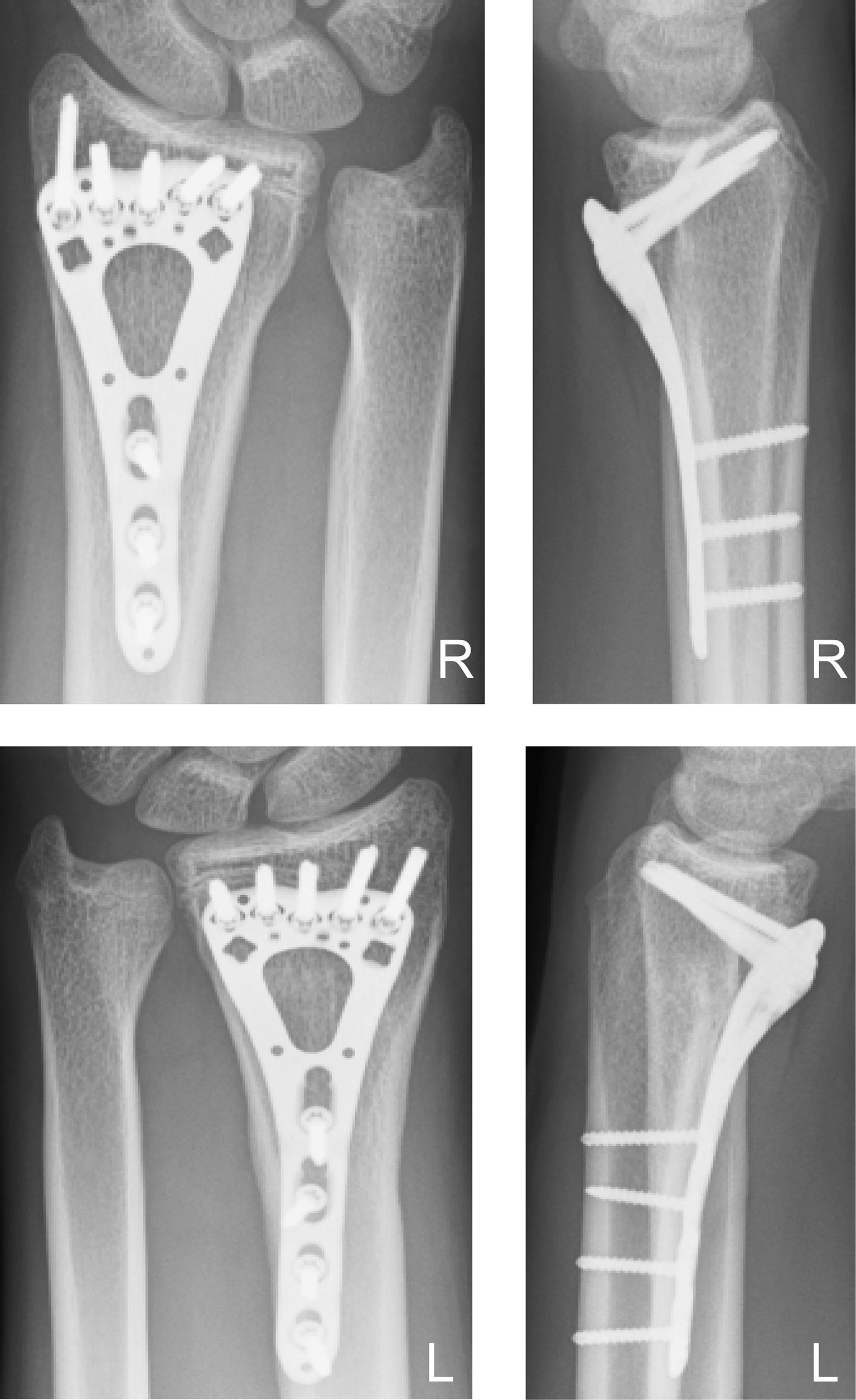
Follow-up imaging case 2. Radiographs showing healed fractures 3 months postoperatively

A healthy 18-year-old man was admitted to the emergency department after falling off his motocross bike at high speed onto both hands. The physical examination showed painful swelling of both wrist without neurovascular impairment. CT scan within the shock room demonstrated a displaced transitional fracture of the distal radius on both sides (Fig. 3). An open reduction and internal plate fixation of both fractures was performed through a volar approach (modified Henry approach): see above for details. On both sides, a Variable Angle LCP Two-Column Volar Distal Radius Plate 2.4 mm (DePuy Synthes, CH-4528 Zuchwil, Switzerland) was attached to the distal radial shaft ending at the anatomic watershed zone of the distal radius under observation from the image intensifiers. Following functional rehabilitation without cast immobilization, the patient demonstrated bilateral equal range of motion of the wrist with radiographic examination showing complete healing of the fractures 3 months post-operatively (Fig. 4). Administration of the Quick DASH (Disabilities of the Arm, Shoulder, Hand) produced a score of 13.6. Removal of osteosynthesis material is planed after 1 year.

### Case III

**Fig. 5:**
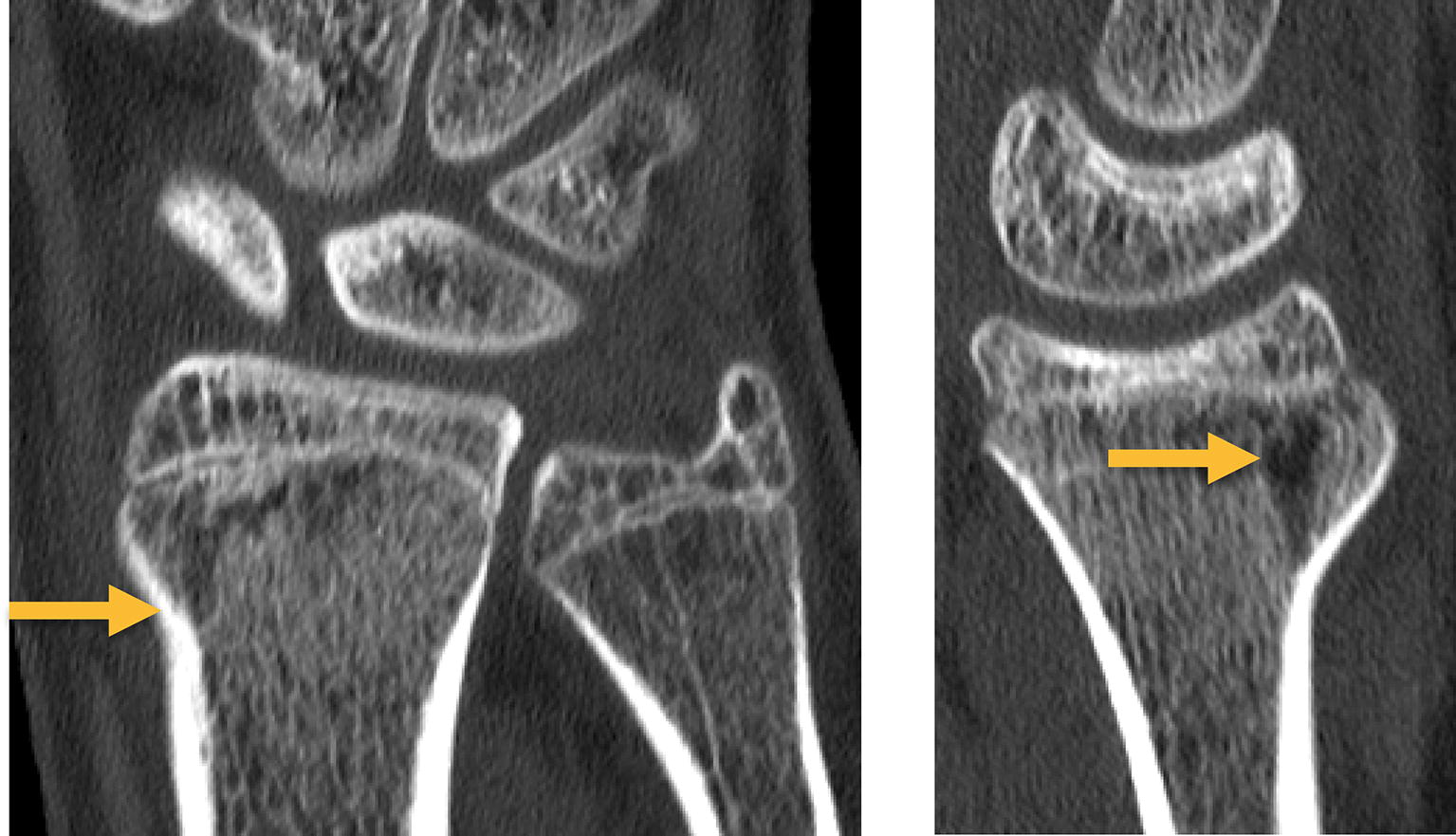
Diagnostic imaging case 3. CT scan demonstrating a minimal displaced transitional fracture of the right distal radius (arrow)

**Fig. 6:**
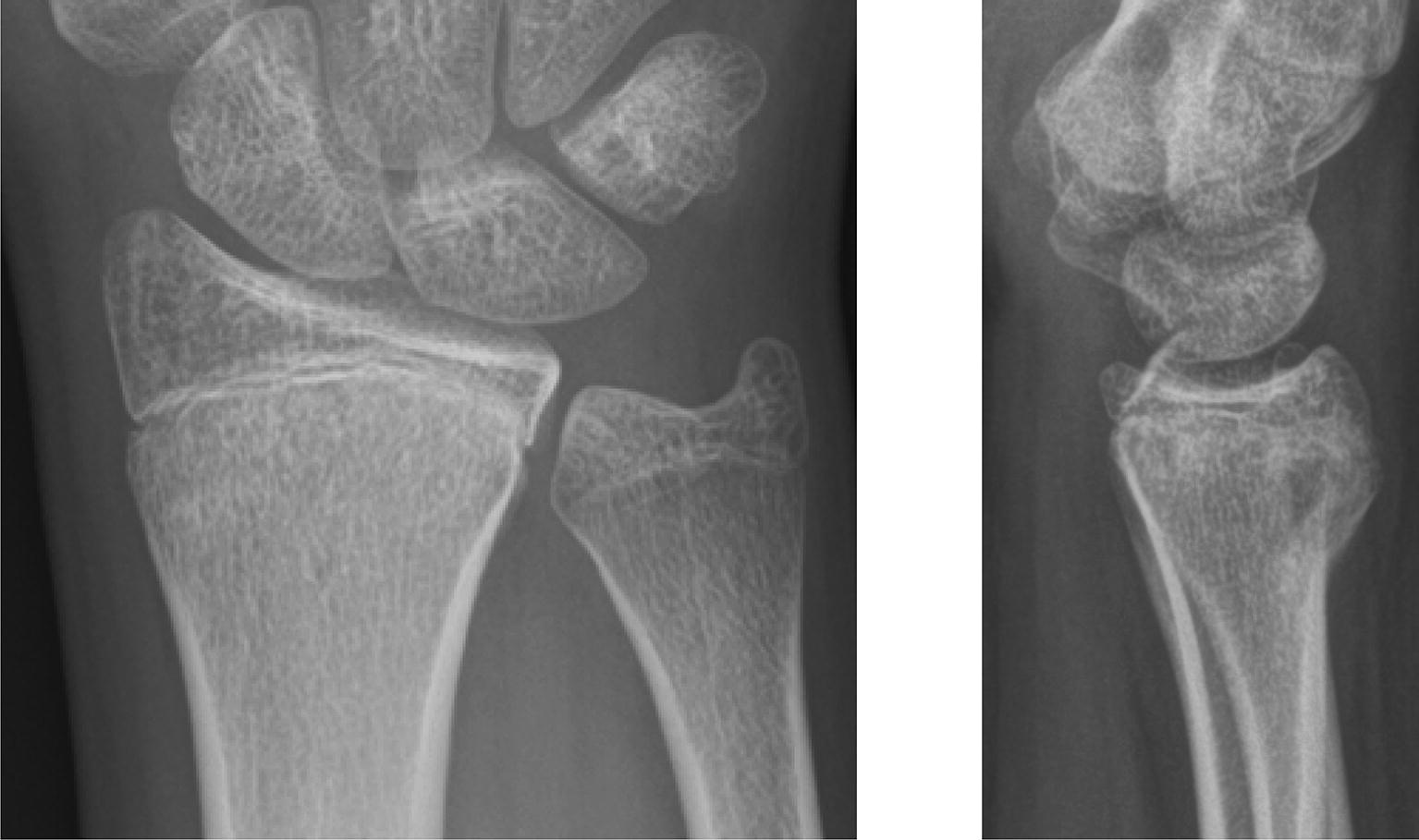
Follow-up imaging case 3. Radiograph showing healed fracture 3 months after trauma

A healthy 17-year-old woman injured her right wrist playing soccer. Physical examination showed marginal painful swelling of the right wrist without neurovascular impairment. Radiograph and the subsequent CT scan demonstrated a minimally displaced transitional fracture of the distal radius (Fig. 5). A non-operative treatment approach with immobilization in a forearm cast for 4 weeks was initiated. Three months postoperatively the patient showed bilateral equal range of motion of the wrist with radiographic examination demonstrating a healed fracture (Fig. 6). At that time, administration of the Quick DASH (Disabilities of the Arm, Shoulder, Hand) resulted in a score of 0.

## Discussion

Transitional fractures are a special type of fracture in adolescents with partial closure of the epiphyseal growth plate, that show different specific fracture patterns compared to fractures in children with wide open physis [10]. Transitional fractures are most commonly reported in the distal tibia [10]. Depending on the number of fragments, the transitional fractures of the distal tibia are divided in two-plane, three-plane I or three-plane II fractures [17]. Theoretically, these fractures could occur in any other physeal growth plate of the body. However, locations other than the distal tibia are very rare and only a few individual case reports are documented [18–21]. Until today only five case reports of transitional fractures of the distal radius have been published [11–16].

The physiology of the closure of the epiphyseal growth plate in general, and closure of the distal radius in particular, as well as the forces acting at the distal radius need to be reviewed to discuss the different treatment options of this rare injury.

The distal part of the radius has a quadrilateral cross-section including the metaphysis and the epiphysis [22]. According to the biomechanical model of Rikli and Regazzoni, the distal forearm is divided into three columns: the radial, the intermediate and the ulnar column [23, 24]. A biomechanical study evaluating the distribution of forces transmitted across the human radioulnocarpal joint under physiologic conditions and was able to confirm the existence of two main force transmission centers: one at the intermediate column opposite the proximal pole of the scaphoid, and the second at the ulnar column opposite the lunate [25]. Furthermore, it was demonstrated that the majority of the load (up to three-fourths) was transmitted through the ulnar column [25, 26]. During daily living activities, wrist movements can generate loads of approximately 100 N, while finger flexion produces median forces of 250 N [22, 27]. Another biomechanical study showed that, depending of the wrist position, for each applied 10 N grip force, a force of 26 to 52 N is transmitted to the distal radius [28]. A 2015 updated overview of normative values for grip and pinch strength for 6- to 19-year-old patients demonstrated that grip and pinch strength generally increased with age, while males showed greater strength than females, while hand dominance was not a significant factor affecting hand strength [29]. A grip force of 450 N leads to an applied load of 2410 N on the radial metaphysis in adults [30]. It is presumed that a load greater than 2500 N is required to break the distal radius [31].

Comprehensive understanding of the physiological closure of the physeal plate is essential to make the diagnosis and select an appropriate therapy in the treatment of transitional fractures. Physeal plate maturation in general can be divided into three stages: the growth stage, with balanced processes of proliferation and mineralization; the resting stage, with suspended proliferation; and the closure stage, with final mineralization of the physeal plate, starting from the metaphyseal and subsequently also from the epiphyseal region [32]. Specific information about the dynamics of maturation of the physeal plate is only present for the distal tibia. In contrast, relatively little is known about the epiphyseal closure of the distal radius. A study of sequential MRI investigations of 22 adolescents demonstrated that mineralization of the physeal plate of the distal radius begins centroradial and ends with a small dorsoradial limbus [32]. Furthermore, it was demonstrated that closure of the physeal plate of the distal radius takes place during late adolescence (15 to 18 years) and occurs within less than a year [32]. The study authors concluded that this may help to explain the rare occurrence of transitional fractures of the distal radius. As a consequence of only partial mineralization of the physeal plate, the forces applied during a traumatic event will be transferred by the already mineralized portion of the physeal plate to the yet to be mineralized portion of the physeal plate, resulting in fracture [10].

The overall prognosis for transitional fractures is good. The physiological process of epiphyseal growth plate closure already in progress, will be accelerated due to the trauma and the subsequent increased hyperemia, so that maturation of the affected physeal plate ends earlier than the one of the opposite side [33]. As longitudinal growth during the closure stage of physeal plate maturation has already been completed, clinically relevant growth disturbances are unlikely [33, 34]. Therefore, the remaining remodeling potential of these fractures is very limited.

Transitional fractures are common in the distal tibia. Their specific stereotype fracture pattern (two-plane, three-plane I or three-plane II fractures) and management are well documented in the literature [10, 17, 33, 35].

Fractures of the distal radius, unlike those of the distal tibial, are typically caused by axial rather than torsional forces, which affect the fracture pattern. In this respect, it can be assumed that the specific fracture pattern of transitional fractures of the distal radius could vary from those of the distal tibia.

There is a lack of data concerning diagnosis and optimal treatment of transitional fractures of the distal radius.

Non-operative and surgical treatment options are described. Non-operative treatment (immobilization in a forearm cast for at least 4 to 6 weeks) of transitional fractures of the distal radius was recommended in the published case reports for non- or minimally (less than 2 mm) displaced fractures or if anatomical reduction and maintenance of reduction could be achieved by closed manipulation [11–15]. Mingo-Robinet et al. described in their case report an open reduction and internal fixation treatment with two screws after a failed closed reduction attempt [16].

A study conducted by Kraus et al. found that physeal plate closure of the distal radius occurs during late adolescence [32], suggesting that the age range of male patients from the aforementioned studies [11–16] of 13 to 15 years was too young, as the distal radius physeal plate had yet to close. In our case series, patient ages ranged from 16 to 18 years, including a gender ratio of two males to one female. None of the fractures of the previously published reports were evaluated by CT scan. This may be due to the fact that only non- or minimally displaced fractures were described. In contrast, all of the seriously displaced fractures in the presented case series were evaluated by CT scan with multiplanar reconstruction in accordance with literature recommendations [36]. Furthermore, we are presenting extremely rare cases, both a transitional fracture of the distal radius combined with a transitional fracture of the distal ulna and a simultaneous both-sided transitional fracture of the distal radii.

The commonly accepted treatment goal in transitional fractures is the anatomic reduction of the articular surface [10–17, 20, 33, 34], as longitudinal growth at the time of physeal plate maturation is complete, the remodeling potential of these fractures is limited [17, 33, 34]. We suggest CT scan with multiplanar reconstruction for displaced transitional fractures for a closer understanding of the fractures and to allow for better treatment planning [34, 36, 37]. Non-operative treatment (immobilization in a forearm cast for at least 4 to 6 weeks) of transitional fractures of the distal radius is recommended only for non- or minimal (less than 2 mm) displaced fractures or if anatomical reduction and maintenance of reduction could be achieved by closed manipulation. Significantly displaced and unstable transitional fractures of the distal radius require open reduction and internal fixation to restore anatomic joint conditions. Different fixation options have been described, including the use of K-wires and lag screws [10, 16, 34]. However, the presented cases demonstrate that volar plate fixation followed by functional rehabilitation is a valuable treatment option in significantly displaced transitional fractures, especially in older adolescents. This is because longitudinal growth at the time of fracture occurence has already been completed and clinically relevant growth disturbances are unlikely [13, 33, 34]. This is further supported by the fact that Quick DASH Scores in both of our patients treated with volar plate fixation 3 month postoperatively was less than 15 points, signifying an excellent functional outcome.

## Conclusions

Since most of the patients with transitional fractures of the distal radius are physically active and participate in sports, the key to successful treatment of transitional fractures of the distal radius is a proper understanding of the fracture, achieved by CT scan with multiplanar reconstruction, to reach anatomic reduction of the articular surface followed by functional rehabilitation when possible to achieve a positive functional outcome.

## Data Availability

The raw data used in the analyses of this study are available in the authors’ database.
